# Reservoirs of Getah virus: an update review

**DOI:** 10.3389/fvets.2026.1901068

**Published:** 2026-07-17

**Authors:** Jia-Xi Li, Hua-Hua Kang, Sheng-Nan Chen, Xia Zhou, Wei Wang, Yong-Song Li, Zhu-Qi Hu, Xiao-Ming Zhou, Shao-Lun Zhai

**Affiliations:** 1 Institute of Animal Health, Guangdong Academy of Agricultural Sciences, Guangdong Provincial Key Laboratory of Livestock Disease Prevention, Scientific Observation and Experiment Station of Veterinary Drugs and Diagnostic Techniques of Guangdong Province, Guangzhou, China; 2 School of Life sciences, South China Normal University, Guangzhou, China; 3 Laboratory of Animal infectious Diseases and Molecular Immunology, College of Animal Science and Technology, Guangxi University, Nanning, China; 4 Guangzhou Sino-Science Gene Testing Service Co.,Ltd., Guangzhou, China; 5 Healvet Medtech GZ Ltd., Guangzhou, China; 6 V.L Healthnet Services Ltd., Lusaka, Zambia; 7 Guangzhou Huadu Animal Health Supervision Center, Guangzhou, China

**Keywords:** Getah virus, new emerging reservoirs, reservoir diversity, reservoir expansion, traditional reservoirs

## Abstract

Getah virus (GETV) is a mosquito-borne RNA virus whose cross-species transmission poses a potential threat to both animal and public health. Studies have revealed that the reservoir range of GETV is broad, ranging from primary vectors (e.g., mosquitoes) and key amplifying hosts (e.g., horses and pigs) to a variety of newly identified hosts such as foxes, cattle, red pandas, and squirrels. Notably, although no confirmed clinical cases in humans have been reported to date, serological evidence from healthy individuals and febrile patients in tropical and subtropical regions of Asia and Oceania suggests possible subclinical or past infections. Furthermore, the detection of GETV in wastewater treatment plants and its contamination of veterinary vaccines underscore the risks of environmental and iatrogenic transmission. These findings demonstrate the broad host diversity and multiple transmission routes of GETV, highlighting the need to monitor and mitigate its cross-species transmission risks. This review comprehensively summarizes the known reservoirs of GETV, with the aim of informing future research and control strategies.

## Introduction

**Figure 1:**
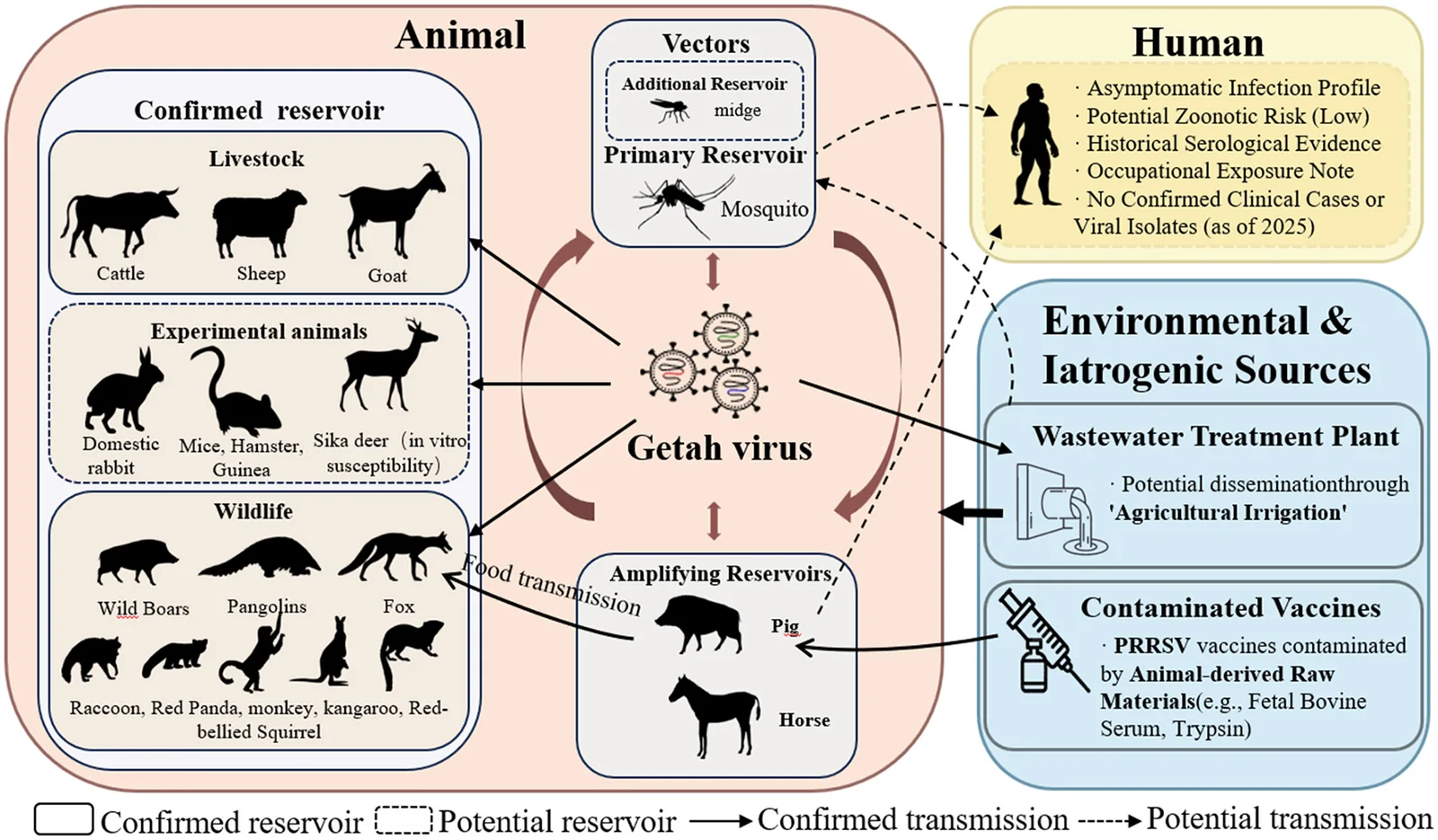
GETV transmission network.

Getah virus (GETV) is a mosquito-borne zoonotic pathogen belonging to the genus *Alphavirus* of the *Togaviridae* family (1, 2). GETV virions are spherical, enveloped particles approximately 60–70 nm in diameter, with a densely packed icosahedral nucleocapsid. The genome consists of a single-stranded, positive-sense RNA of about 11.7 kb in length, which includes two open reading frames (ORFs): ORF1encodes the non-structural proteins (nsP1–nsP4), and ORF2 encodes the structural proteins (capsid, E3, E2, 6K, and E1) (1). Its transmission is maintained through an enzootic “mosquito-vertebrate-mosquito” cycle, with mosquitoes serving as the primary vectors and horses and pigs identified as key vertebrate hosts (1). GETV infection can cause fever and edema in horses, reproductive disorders in sows, and piglet mortality (3, 4). Notably, piglet diarrhea is a common clinical manifestation in affected pig herds, often leading to significant economic losses due to dehydration, growth retardation, and increased mortality (1, 5). Since the first case of horse infection was reported in Japan in 1978, GETV antibodies and/or antigens have been detected in a wide range of animals including cattle, foxes and squirrels, as well as humans (1, 4, 6, 7). Notably, beyond animal hosts, GETV has also been detected in wastewater and contaminated veterinary vaccines, raising concerns about its possible transmission through vaccination (8, 9). The primary objective of this review is to systematically update and summarize the reservoirs of GETV (Figure 1).

## Traditional reservoirs

### Arthropod

#### Mosquitoes

GETV was first isolated in 1955 from *Culex tritaeniorhynchus* mosquitoes in Kuala Lumpur, Malaysia (prototype strain MM2021) (10) and is primarily transmitted by mosquitoes. The most important vectors belong to the genus *Culex*, including *Cx. tritaeniorhynchus, Cx. gelidus, Cx. annulus, Cx. pseudovishnui, Cx. vishnui*, and *Cx. fuscocephala*. Within the genus *Anopheles, An. sinensis* and *An. vagus* are major vectors. *Aedes*-associated transmission involves *Ae. japonicus, Ae. albopictus, Ae. vexans nipponii* and *Aedes spp*. in the Russian Far East. Additionally, *Armigeres subalbatus* is also a key vector. Experimental studies have confirmed the susceptibility of *Ae. japonicus, Ae. aegypti, Cx. pipiens pallens*, and *Tx. bambusa* to GETV, although their roles in natural transmission cycles require further investigation (11–23). GETV exhibits broad vector adaptability and cross-regional distribution, highlighting its ecological plasticity and potential public health risks.

#### Midges

Beyond mosquitoes, midges (Culicoides spp.) also serve as GETV vectors, with sporadic detections in China and Eurasia. GETV was isolated from midges in Yunnan (2013) and Xinjiang (2016), and these strains show high genetic homology with mosquito and vertebrate isolates (1, 23–25). Specifically, the Yunnan isolate SZC30 clustered within Group III alongside GETV strains from mosquitoes and pigs in China, Korea, and Japan, with nucleotide and amino acid homologies of >98.0% and >98.9%, respectively, suggesting midges as potential local vectors (26, 27).

The ecological role of midges warrants attention, particularly in southern China where warm, humid conditions favor year-round vector activity (26, 28). Many Culicoides species exhibit low host specificity, feeding on both domestic animals and wildlife, positioning them as potential “bridge vectors” between wildlife and domestic populations (29, 30). Thus, midges may act as additional vectors in GETV circulation among pigs, cattle, and wildlife in southern China, especially in peridomestic livestock farms where both midges and mosquitoes are abundant (26, 28).

The detection of multiple arboviruses (e.g., Bluetongue virus, Akabane virus) in Culicoides worldwide underscores their vector competence (31–33). Although GETV has not been isolated from midges in all endemic regions, the isolation of SZC30 from Yunnan—showing >98.0% nucleotide and >98.9% amino acid homology with mosquito and pig isolates (26)—together with the established role of Culicoides as vectors for other arboviruses, supports the hypothesis that midges contribute to GETV transmission. These findings call for integrated vector surveillance including both mosquitoes and midges, particularly in tropical and subtropical China (28). Additionally, a Xinjiang midge-origin strain shares 98% similarity with a Japanese isolate from 2005, suggesting possible introduction through imported livestock; thus, quarantine measures in livestock trade should be strengthened (1).

### Vertebrate

#### Horses

GETV is recognized as a significant pathogen in horses. Since the 1970s, six large-scale outbreaks have been reported in horse populations in Japan and India (34). In 2018, China reported its first confirmed case of GETV infection in horses (35). Clinical manifestations of horses infected by GETV include fever, accompanied by a range of symptoms such as urticaria, edema in the hind limbs, swollen lymph nodes, abnormal gait, abdominal pain, jaundice, and scrotal edema (13).

#### Pigs

Substantial evidence has established the pig as a major reservoir and amplification host for GETV. Experimental infection can induce reproductive disorders in pregnant sows, primarily manifested as abortion, stillbirth, mummified fetuses, and decreased reproductive performance. Newborn piglets may present with diarrhea, neurological symptoms such as tremors and hind-limb paralysis, and subcutaneous congestion; in severe cases, the infection can be fatal. Moreover, GETV has been frequently isolated from naturally infected cases (36–38). During the outbreak at a pig farm in Hunan, China, the GETV infection rate rose sharply from 32.65% to 98.33%, confirming the high infectivity of GETV (4). Notably, the porcine and horse strains isolated during the 2014–2015 Japanese epidemic are genetically homologous, providing direct evidence for cross-species transmission (39). These findings collectively establish pigs not only as important amplification hosts for GETV but also as critical components in its maintenance and transmission cycle.

### Rodents

Mice, hamsters, and guinea pigs are commonly used experimental models in GETV research (38, 40–42), but evidence of natural infection in wild rodents is limited. To date, the virus has been detected only in the red-bellied squirrel in Fujian, China (7). Although laboratory studies have confirmed rodent susceptibility (38, 40–42), their natural reservoir competence, prevalence, and specific role in endemic areas remain unclear and further research is needed.

### Human

GETV is considered a potential zoonotic pathogen. Serological studies have shown that it is widely prevalent in Asia and Oceania. Individuals with evidence of GETV infection have been reported in Malaysia, China, Australia, and the former Soviet Union, suggesting historical infections (1, 43, 44). However, as of 2025, no confirmed clinical cases or viral isolates from human samples have been reported, and the possibility of serological cross-reactivity with other alphaviruses cannot be ruled out. Therefore, GETV remains primarily an animal pathogen, and current evidence supports it as a potential zoonotic agent with a low or asymptomatic infection profile, rather than an established human pathogen. Nevertheless, given its outbreaks in domestic animals and the known human pathogenicity of related alphaviruses (45, 46), the potential risk to occupationally exposed groups warrants vigilance. Overall, GETV remains primarily an animal pathogen, but its zoonotic potential and impact on public health merit further investigation. The recent global outbreak of Chikungunya virus (CHIKV) highlights the increasing threat of mosquito-borne viruses. In Guangdong, China, 2,940 new local cases were reported within a week (47). Although GETV primarily infects animals, it belongs to the same genus (*Alphavirus*) as CHIKV and shares common mosquito vectors, suggesting a comparable potential for epidemic emergence. This situation underscores the urgency of strengthening collaborative surveillance and control measures against emerging mosquito-borne alphaviruses, including GETV. Consequently, the CHIKV epidemic serves as a critical reminder of the threats posed by the cross-species transmission of viruses like GETV.

## New types of reservoirs

In addition to traditional reservoirs such as mosquitoes, horses and pigs, a range of new hosts has been identified, highlighting an increasingly complex transmission network for the virus.

### Ruminants

Recent studies indicate an increasing prevalence of GETV infection among ruminants, highlighting a growing concern for animal husbandry. In 1969, GETV antibodies were first reported in cattle in Queensland, Australia, although technical limitations at the time precluded the exclusion of cross-reactivity. After 2015, serological evidence was recorded, and the virus was first successfully isolated from cattle in 2018 (48–50). In 2017, GETV detection rates in goats and sheep in northwest China reached 11.7% and 10.0%, respectively (51). Although natural infection has not been documented in wild sika deer, their testicular cells have been shown to be susceptible to GETV infection *in vitro*, indicating their potential role as hosts (52). Collectively, these findings suggest that ruminants may play a significant role in the transmission of GETV, warranting further investigation into their contribution to the transmission cycle.

### Poultry

Studies have shown that GETV can cause cytopathic effects in chicken embryo fibroblasts (CEF) and lesions on the chorioallantoic membrane (CAM) (53, 54), indicating that poultry species are susceptible to infection. Subsequent serological investigations in Yunnan province revealed low GETV detection rates (2% in chickens and 6% in ducks) with relatively low antibody titers (49). These findings suggest that poultry can be infected and may contribute to the transmission cycle, they likely function as secondary or incidental hosts rather than primary reservoirs.

### Rabbits

Under experimental conditions, rabbits are susceptible to GETV (40). This susceptibility was confirmed in a natural setting when the first GETV strain was isolated from domestic rabbits in Jiangxi Province, China, in 2018 (55). This finding confirms the domestic rabbit as a natural host of GETV and validates its potential role in the ecology of GETV.

### Wildlife

GETV exhibits a broad host range within wildlife populations. For instance, the GETV infection rate in wild boars in Japan was reported to be 16.0% (56). Furthermore, a study in Shandong, China, demonstrated that foxes acquired GETV infection through the consumption of contaminated pig organs (57), confirming the risk of foodborne transmission. GETV has also been detected in a variety of wild animals, including raccoons (58), red pandas (59), pangolins, monkeys, and kangaroos (13, 60). These reports underscore the multi-host capacity of GETV and implicate both biological vectors and anthropogenic activities, such as the wildlife trade and habitat encroachment, in its transmission, highlighting the integral role of wildlife in the virus's ecological cycle.

### Biological products

GETV-contaminated veterinary vaccines represent an emerging concern for the swine industry. In 2020, the GETV-V1 strain was first identified as a contaminant in commercially available porcine reproductive and respiratory syndrome virus (PRRSV) vaccines in China. Subsequent studies further confirmed these findings and traced the likely source to animal-derived raw materials such as trypsin and fetal bovine serum, used in the vaccine production process. The use of such contaminated vaccines can cause reproductive failures in sow herds and piglet mortality. These findings necessitate stricter testing protocols for vaccines and optimized production processes to mitigate such risks (9, 61, 62).

These contamination incidents reveal gaps in vaccine biosecurity quality control. Animal-derived materials (e.g., FBS and trypsin) pose risks of adventitious virus contamination, including GETV. Therefore, routine RT-PCR screening of raw materials for GETV is essential. Furthermore, adopting serum-free or chemically defined culture media, along with gamma irradiation of trypsin, could significantly reduce such risks (63). These findings call for stricter testing protocols and optimized production processes. From a One Health perspective, ensuring vaccine safety protects animal health and prevents iatrogenic GETV spread, safeguarding both livestock production and public health.

### Environmental samples

Research on the environmental distribution of GETV remains limited. To date, metagenomic analyses provide the primary evidence, having detected GETV in wastewater, with its average abundance in treatment plant effluent reaching 3.5 × 10?7, a level significantly higher than that of other viruses such as pseudocowpox virus. The persistent detection of GETV in this context suggests a potential for dissemination through wastewater reuse practices, notably agricultural irrigation, which represents a potential public health concern (8). Experimental studies have investigated whether GETV is transmissible via aerosols, demonstrating that this route requires a high viral titer (≥107TCID50/ml). However, the nasal viral load generated during natural infection is typically insufficient to achieve this threshold. Mosquitoes remain the main mode of transmission, while aerosols play a negligible role in natural transmission (13).

## Discussion

The expanding host range and environmental detection of GETV raise important ecological and epidemiological questions. Climate warming may facilitate the geographic expansion of key mosquito and midge vectors, potentially increasing GETV transmission risk in temperate regions (64). Recent genomic surveillance has revealed rapid evolution in GETV, with multiple positively selected amino acid sites in the E2 protein, indicating ongoing adaptive changes. This trend is further supported by a 2024 GIII variant from Henan, which carries four unique mutations in nsP3 and E2 and exhibits markedly enhanced virulence compared to earlier isolates (5, 65). However, direct evidence linking specific mutations to expanded reservoir competence remains limited.

A critical caveat to this expanding reservoir list is the inherent bias in surveillance efforts. The apparent prominence of pigs, horses, and cattle may overstate their ecological importance, as these species are subject to intensive veterinary monitoring for commercial and health reasons. In contrast, wildlife including rodents, birds, and companion animals, remain considerably under-sampled, potentially masking their roles in GETV transmission. Similarly, the scarcity of human detections may partly reflect diagnostic neglect: febrile illnesses are rarely screened specifically for GETV, and serological cross-reactivity with other endemic alphaviruses (e.g., Ross River virus and CHIKV) complicates data interpretation.

Beyond these surveillance-related explanations, however, a more perplexing biological paradox remains: GETV exhibits a remarkably broad host range—encompassing mosquitoes, pigs, horses, ruminants, wildlife, and even experimental evidence of susceptibility in multiple mammalian cell lines—contrasted with the complete absence of confirmed clinical human cases. This discrepancy may be explained by several nonexclusive hypotheses. First, GETV might have inherent species barriers at the level of viral entry, replication efficiency, or innate immune evasion in human cells, despite its apparent ability to bind to ubiquitously expressed receptors such as MXRA8 (which is utilized by multiple alphaviruses) (66). Second, even if human infection occurs, it may be clinically silent or result in only mild, nonspecific febrile symptoms that are rarely diagnosed, particularly in regions where other febrile illnesses (e.g., dengue, CHIKV, and Ross River virus) are endemic. The seropositivity detected in healthy individuals and febrile patients across Asia and Oceania supports this subclinical or oligosymptomatic infection scenario (67, 68). Third, the lack of systematic active surveillance for GETV in human febrile illness cohorts, combined with potential serological cross-reactivity with other alphaviruses, may have led to substantial underdetection (17, 67, 68). Lastly, human exposure routes may also differ from those of domestic animals: while pigs and horses experience intense mosquito feeding in peridomestic settings, human–vector contact is likely more intermittent or involves lower viral inocula, which could partly explain the absence of documented clinical cases (1, 4, 13, 43). Nonetheless, the recent emergence of a highly pathogenic GIII variant in pigs with enhanced virulence underscores the need for continued vigilance (65). The host-range breadth of GETV, even without documented human disease, should not be dismissed; it provides a stark reminder that cross-species adaptation can occur unpredictably, and proactive surveillance in high-risk occupational groups (farm workers, veterinarians, and slaughterhouse personnel) is warranted.

Addressing these gaps through integrated, unbiased vector and host surveillance—particularly in migratory birds and companion animals—will be essential for assessing the emergence potential of GETV within a One Health framework.

## Summary

As of 2025, 70 years have passed since the initial report of GETV from mosquitoes in 1955. During this period, the known reservoirs of GETV have expanded considerably, encompassing not only mosquitoes and domestic animals but also a wide range of wildlife and humans. Its detection in unconventional sources such as contaminated vaccines and wastewater environments further demonstrates its ecological plasticity. This expanding host range and environmental persistence underscore the growing significance of GETV as a zoonotic agent with the potential to infect more species. A comprehensive understanding of its presence across animals, humans, and the environment is therefore crucial for developing effective “One Health” strategies to mitigate its threat (69).
